# Supplementary material for: Patterns of Intron Gain and Loss in Fungi
Source: PLoS Biol. 2004 Nov 30;2(12):e422. doi: 10.1371/journal.pbio.0020422 (PMC532390; doi:10.1371/journal.pbio.0020422)
Supplement: Table S1 — Also available at http://genes.mit.edu/NielsenEtAl/. (4.3 MB ZIP). [file pbio.0020422.st001.zip › NielsenEtAl/html/834.html]

AN4310.1.NCU01847.1.MG02514.1.FG01544.1


```
 CLUSTAL W (1.82) Multiple Sequence Alignments - Introns Inserted


Sequence 1: NCU01847.1	1487 aa
Sequence 2: FG01544.1	1112 aa
Sequence 3: MG02514.1	1097 aa
Sequence 4: AN4310.1	978 aa
Alignment Length: 1510 aa
Number Identitical Residues: 404 aa
Alignment Score (without introns) 20455


MG02514.1 	---------~-------MSKVE0ASAPSKG-KAPK--------------SRVT1SSG---
NCU01847.1	MSAPTDSNV0SKHATVEPAPES~PTTARPF-ELDDDEAQENVPLGTDTTTTAT~TQNNTS
FG01544.1 	---------~--------MRIQ~ASTGSQF-KLDN-------------YSTFL~RSN---
AN4310.1  	---------~-------MLPKH~RLHSVRTR----------------------~------
          	                                                            

MG02514.1 	------------PLIAKH0PWVSFLRRPDVAQSATLAGTTRAGGDHLNTD1IYWQSQPVT
NCU01847.1	GSDENPPAKPPRPLTEEQ~KNQQILKEAFPSIDLTVIKAVLRASRGQIEP~AFNALLEMT
FG01544.1 	--------------LALR~LGFSYFRFGLTSVFTHLYGALLRP-----FP~CF-TFLRLS
AN4310.1  	------------------~-------------------------------~---------
          	                                                            

MG02514.1 	DPIRSWCDIG~HRDTCESPLALFYD~V---------------------------------
NCU01847.1	DPDAVQNDDV~PPPPPPRPAAQPGG~PTSTERSQLEADEIYARQLAEHFESTAAYEARTA
FG01544.1 	LVMSPLLDPI~TLPTYPLPQHCCTH0P---------------------------------
AN4310.1  	---RSFFSDW2RPRSVPLSRTAAQK~P---------------------------------
          	       .      .   .                                         

MG02514.1 	----------KTTTR~--------------------------------------------
NCU01847.1	NRGTPGSSREPTAAR~GPHMPPRHRQETGLKPNELYDDREHSFIDDDLPVIKESLRKGFI
FG01544.1 	-------SRAPTPQD0--HTP---------------------------------------
AN4310.1  	---------------~--------------------------------------------
          	                                                            

MG02514.1 	----DINTCFFVS-RKHPRIFPP-------------------------------------
NCU01847.1	ETQSKVNSWFTTIKKKIDETFDEDENERRQNESNAFMGRPTRNQQRRSADYDRYDADPAI
FG01544.1 	----QVHSLLTLSSRKFGKQIQK-------------------------------------
AN4310.1  	---------------DIRRQFSQ-------------------------------------
          	               .  . :                                       

MG02514.1 	---------------~----------------------------------ISLEVFAGKR
NCU01847.1	LSDDFAGMKFNNDGT1PYTAQSTHQQLYGNPHAFRPPPPSKSPKSSDGRKVSFRDTVEDI
FG01544.1 	---------------~--------------------------------RQLEVPEYAASF
AN4310.1  	---------------~-----------------------------------RLKTLVDKK
          	                                                    .   . . 

MG02514.1 	HLTSSFQH0LIKRLSATPVLGA--------------------------------------
NCU01847.1	SAYDASPR~MQPKDSAAAAPGSSSAAGSATKTSKWQPLSSVDPNPIADNDPFSLGDSDDE
FG01544.1 	VNYKALKK0LIKKLSATPTLTS--------------------------------------
AN4310.1  	RVAQSRTV~TKPKAPAN-------------------------------------------
          	   .:       : .*                                            

MG02514.1 	-------------------------QNDIHHTAGT-------LDAQGLLQANKAT~FF--
NCU01847.1	FEQGHRRNQSGSSQIKMEDNTATDADHERLRQATADAMADSLVEGDKAKKSEQAK2KFGK
FG01544.1 	-------------------------QNDILRSATP-------VDSQAALQANKAT~FF--
AN4310.1  	--------------------------------------------LVALLEGFQ--~----
          	                                                 :. :       

MG02514.1 	---------------------FQL0ERELEKVNAFYLQKEAE0-----------------
NCU01847.1	QIQKRQLEVPEYAASFVNYKALKK0DRELDKVNACYVQKEAE~LKIRLKTLLDKKKALRS
FG01544.1 	---------------------FQL0ERELDKVNAFYMQKEAE0LKIRLKTLLDKKKVLQS
AN4310.1  	----------------------QF~DGDLNKL----------~-----------------
          	                      :  : :*:*:                            

MG02514.1 	------------------------------~QFVEINGTAFSKILKKWDKTSK~SKTKEL
NCU01847.1	RSGGTSRRSTKFTTLQEGFQQFVNDLNKLQ~QFVEINGTAFSKILKKWDKTAK~SKTKEL
FG01544.1 	RQG-ISRRSAKFTTLEEGFQQFATDLNKLQ~QFVEINGTAFSKILKKWDKTSK~SKTKEL
AN4310.1  	-----------------------------Q0QFVEINETAMSKILK------K0SRMKEL
          	                             . ****** **:*****      * *: ***

MG02514.1 	YLSRAVEVQPFFNATVISELSDQATTSLQELGAWADGDNVTFSDHR--DHIVTSQNLLGT
NCU01847.1	YLSRVVEKRPAFNPTVISELSDQATTSLQELGAWADGDNVSFETRPDHDHVVSTQHLLGT
FG01544.1 	YLSRAVEVQPFFNATVISELSDQATTSLQELGAWSDGIQVNFQSG----HVVTSQHFVGT
AN4310.1  	YLHRAVEVQPCFNRDVLRDLSDRATTARIELEAWAEGENIQFDTTR-PTDRTTTFQGFGS
          	** *.** :* **  *: :***:***:  ** **::* :: *.      . .:: : .*:

MG02514.1 	DEGDVDTLLLETVISGNLDSLKDLLTRMRSTSPDPGATSDMDVPLKERITRTFLASINEG
NCU01847.1	DEGDADTLLLDTVLSGNIDSLKDMLDRMKATAAPDGSA---DVSLAERVTRTFLASINEG
FG01544.1 	DEGDADTLLLDTVITGNLESLKDLLLRMRS-ANEAGDG---DSSLMERITRTFLAAISEA
AN4310.1  	EEEDLDLQVLQSANSGNLYALREWADKLQK----SGDS-------RERATRTFLAAISEY
          	:* * *  :*::. :**: :*::   :::      *          ** ******:*.* 

MG02514.1 	PQASLEVLLNTGLVDIQSEDDINERNCLHQATIYSNSFVLQYGLSKGVSVSRTDVYGRVP
NCU01847.1	SLEALKVLLDTGLVDIQSWDDINERNCLHQAAIYGNSFVLEYGLSKGVDAERTDAYGRVP
FG01544.1 	PQESLRVLLDTGLVDLHSYDDINERNCLHQAAIYGKQYVLEWGLSVNVVVDRTDVYGRVP
AN4310.1  	SDDVLAVLLETGLVDIFAEDDINERNCLHEAAISGREFVLKAGLEAGVQVSRTDVYGRIP
          	.   * ***:*****: : **********:*:* ...:**: **. .* ..***.***:*

MG02514.1 	LHYASMHGRLDMLA~ALLEA1GPKTIDLIDHDNFTPLIHAIIHGHYECVNLLLLNSARID
NCU01847.1	LHYASMHGRLDMID~ALLNA1SPKTINLIDHDNFTPLVHSIVRNHLECVGRLLERSARID
FG01544.1 	LHYASLHGRLEMLK0LAN--~-QRTIDLTDHDNFTPLIHSIIHCHLNCIELLLARSARID
AN4310.1  	LHYACIHGRVGMVQ~QLLAT~DPSTVDIMDHDNFTPLIHSIVKNQLDCAEQLLRNNARID
          	****.:***: *:      : .  *::: ********:*:*:: : :*   ** ..****

MG02514.1 	PISDTDHVPVNLACEHGSAAIVELLLRQGAKIVPDAEGLYPQHLVARSGQTSDLLLLLNQ
NCU01847.1	PVSDTDHVPLNLACQHGSVAIVELLLKHGAKILADAEGLYPQHLVARSGQTPEILVLLKQ
FG01544.1 	PVSDADHVPLNLACEHGSVAVVEMLLKHGANILPDAEGLYPQHLVARAGQTSELLLLLKE
AN4310.1  	PVSESDHIPLNLACQHGSLPIVKMLLERQARLLPDAEGLYPQHMVARASQSPDILLLLKR
          	*:*::**:*:****:*** .:*::**.: *.::.*********:***:.*:.::*:**:.

MG02514.1 	YGADLNQMDKLYGWTPLVHAASEGNVPCLKVLLEVGVDPNILDEKHLPALYYAAWEGHLE
NCU01847.1	YGADLDQIDKLYGWTPLVHAASEGNVPCLQALLETGADPNILDEKDLPAMYYAAWEGHLE
FG01544.1 	FGADLDQIDKLYGWTPLVHSASEGNVDCLQALLKVGVDASIVDEKDLPAMYYAAWEGHLA
AN4310.1  	HGADLNQRDKLYQWTPLFHAASEGCVNCLRTLLELGVDADVKDEKGLAAVYYAAWEGHLE
          	.****:* **** ****.*:**** * **:.**: *.*..: *** *.*:********* 

MG02514.1 	CMKLLSPFNRTKAPSPLVRPIPATPLSLLGSS-------SAPIPMALDP-DAIPVLELPP
NCU01847.1	CMKLLTPAKKEKAASELPPIHIGGALPPMASS-------TAPMPMSLDAIDPIPALELPP
FG01544.1 	CMKLLTPFNTRPRAS---PFMVQPALGPMDSS-------TAPLPMSLDP-DAIPELELPP
AN4310.1  	CMLLLWSHHTESQPSQRPLDILNGLRLHEPSSTIAWQEHTPAEAQDMETVDGIPDLSLPP
          	** ** . :    .*               **: : .. :.. .  ::. * ** *.***

MG02514.1 	PIIPLRRYGHNFLDTKTVVQINFGEGGQQPLMFFHNSKYPASRLTISSKASDLIPKNIIL
NCU01847.1	PIIPLRRYGHNFLDTKTVVQISFEEDSEQPLLFFQDGKYPAARLTISSKSSDLIPKNIIL
FG01544.1 	PIIPLRRYGHNFLDTKTVVQISFGDVDEQPLVFFQDGKYPAARLTISSKVSDLIPKNIIL
AN4310.1  	PIIPLRRYGHNFLDKKVFVQILFDPGS--SISFDQAGRYPAARLTISSKLSELIPRTVML
          	**************.*..*** *   .  .: * : .:***:******* *:***:.::*

MG02514.1 	PFQEDTRLVSFHVDNLDSFTLDFDVFPAYGAKVIAKTVALPSTFRALLSSAGNSCCLPLF
NCU01847.1	PFQEDTRVASFQIDNLESFTLDFEVFPTYGAKVIAKTVALPNIFRALLSSSG-KCCLPLF
FG01544.1 	PFQEDTRIVSFQVDSLETFSLDIDVFPTYGAKVIAKTVALSSIFKGLQGTS--VCCLPLF
AN4310.1  	PIQEDARMISFHVDTLQTFTVDFEIFPTFGSKVIAKTVALPTVFGAEESSAG-SCCLPLF
          	*:***:*: **::*.*::*::*:::**::*:*********.. * .  .::.  ******

MG02514.1 	DPRLRAIGQISFHTQVIKPFRGVPLEISDFETYWKATSQLDGQPSTIVSDSSLSGDFVRL
NCU01847.1	DPRLRAIGQISFHVQIIKPFSGTPLEITDFETYWKATSQFDTNTSTFVTGSSLSGDFVQI
FG01544.1 	DPRLRAIGQISFTVQVIKPFQGKPLEITDFETYWKATSQFNQPTSAIVTGSSLSGDYVRL
AN4310.1  	DPRLRAIGQLRFWFQVIKPYHGDPLEITHFATYWKATSTGESDHSGLVTGSSLSGDHVQL
          	*********: *  *:***: * ****:.* *******  :   * :*:.******.*::

MG02514.1 	HVQQTSDGVPVLWPYWTVSCGNGVAVPVARLKYEQFAIAVAASGPAVFNNVNERVSGIAR
NCU01847.1	YVQHTKDGVPVLWPRWTINCG-GIDVPVSTLTLAQFQTVTAAA--------RNRIN--LS
FG01544.1 	FVQYTSDGVPVLWPRWTIPCA-GLDIPVCRLTLEQFGSMTIRS--------NSRAD--LP
AN4310.1  	FVQLTRDKVPVVHPQYMVKHY-GIDIPICHLTFSEFQAVGGEK--------NVNRPELLQ
          	.** * * ***: * : :    *: :*:. *.  :*               . .      

MG02514.1 	QVASTPPDRISYVHEILATSGVRLQDAFLMLPAGMHVNLQIIYPTAEEDRELGLLGPGAD
NCU01847.1	ELSTYTLDQIADVHRILANIGITLHEALFLLPKGMHVNIQVLYPTADEKAEAKSSTAADD
FG01544.1 	SLINKSSESIAEVYHILATAGVTLQEALALLNPGMHVNLQVLYPTSEE-EKAFSLGPALD
AN4310.1  	FLQAHAADDLALTHRVLAASFLSLREVFENLPVDVNVNISVLYPSAAE-EQALKMTSMTD
          	 :   . : :: .:.:**   : *::.:  *  .::**:.::**:: *  :     .  *

MG02514.1 	MNTFADDVLSVVFDHARAQRAQAPDAVRSVVFSSYNPSLCTALNWKQPNF~PVFLCNDLG
NCU01847.1	VNEFADAILSVVFDHARAQRAERPDSVRSVVFSSYNPTLCTALNWKQPNF~PVFLCNDMG
FG01544.1 	VNVFVDSILNIVFEHARTQRAQSPDVVRSVVFSSYSPRLCTALNWKQPNF~PVFLCNDLG
AN4310.1  	VNTFADAILTVVFDHARVARDKNPEFMRSVVFTSYNANICVALNWKQPNY1PVLLCNDLG
          	:* *.* :*.:**:***. * : *: :*****:**.. :*.********: **:****:*

MG02514.1 	QETADG-------MAPQIG----------------DTDRACGSRTSSIKEAVRTAESNNF
NCU01847.1	REDRKQQQQGSCSKGDGDEDMGGTTAASRREAADERTLQSDGRRTSSIKDVVRTATSNNL
FG01544.1 	REETS-----------GVNDMA----------------LSSGRRSASIKEVVRIAQSNNF
AN4310.1  	QIRDLAR-------NAGTHP----------------DVDSSGRASMSIKESARVAQSNNF
          	:                                      : *  : ***: .* * ***:

MG02514.1 	MGLICCSRLL0DMVPALVDAIKSHGLALVVDKSRERQPDSGG----------------LV
NCU01847.1	MGLICCSRLL0DMVPALVDAIKSHGLALVVDKSGEPEAANSKDSGDKQLGISGALGGLAG
FG01544.1 	MGLICYSPLL0DMVPALVDAIKSHGLALVTDKSSDSPNASP------------------M
AN4310.1  	MGLICRSSLL0NVVPALVATIKELGLVLVADTSDEAEQPETQALSAAG--------SMGV
          	***** * ** ::***** :**. **.**.*.* :    .    .           .   

MG02514.1 	VDRFPRLPNGVDGVLKGNGVLRFNESIDM
NCU01847.1	SDPFPKLPKGVDGLLKSNGVLRFNEYIDV
FG01544.1 	TDPFPRPPKGVDGVLKSHGILRFNDSIDM
AN4310.1  	AELAYRMPEGVNGLMKANGILRFNDMIDM
          	 :   : *:**:*::*.:*:****: **:
```
